# Hot and cold tumors: Immunological features and the therapeutic strategies

**DOI:** 10.1002/mco2.343

**Published:** 2023-08-26

**Authors:** Lianjie Wang, Hui Geng, Yujie Liu, Lei Liu, Yanhua Chen, Fanchen Wu, Zhiyi Liu, Shiliang Ling, Yan Wang, Lihong Zhou

**Affiliations:** ^1^ Department of Medical Oncology and Cancer Institute Shuguang Hospital Shanghai University of Traditional Chinese Medicine Shanghai China; ^2^ Department of Internal Medicine Shanghai International Medical Center Shanghai China; ^3^ Department of Nephrology Shuguang Hospital Shanghai University of Traditional Chinese Medicine Shanghai China; ^4^ Department of the Tumor Research Center, Academy of Integrative Medicine Shanghai University of Traditional Chinese Medicine Shanghai China; ^5^ Department of Medical Oncology Ningbo Hospital of Traditional Chinese Medicine, Zhejiang Province Ningbo China

**Keywords:** cancer, hotness and coldness of tumors, immune characteristics, signaling mechanism, therapeutic strategies, tumor microenvironment

## Abstract

The “hotness” or “coldness” of the tumors are determined by the information of the cancer cells themselves, tumor immune characteristics, tumor microenvironment, and signaling mechanisms, which are key factors affecting cancer patients’ clinical efficacy. The switch mechanism of “hotness” and “coldness” and its corresponding pathological characteristics and treatment strategies are the frontier and hot spot of tumor treatment. How to distinguish the “hotness” or “coldness” effectively and clarify the causes, microenvironment state, and characteristics are very important for the tumor response and efficacy treatments. Starting from the concept of hot and cold tumor, this review systematically summarized the molecular characteristics, influencing factors, and therapeutic strategies of “hot and cold tumors,” and analyzed the immunophenotypes, the tumor microenvironment, the signaling pathways, and the molecular markers that contribute to “hot and cold tumors” in details. Different therapeutic strategies for “cold and hot tumors” based on clinical efficacy were analyzed with drug targets and proteins for “cold and hot tumors.” Furthermore, this review combines the therapeutic strategies of different “hot and cold tumors” with traditional medicine and modern medicine, to provide a basis and guidance for clinical decision‐making of cancer treatment.

## Graphical abstract

The characteristics of coldness and hotness tumors are determined by the information of cancer cells and tumor microenvironments, which significantly affects cancer patients’ clinical efficacy. Different therapeutic strategies for cold and hot tumors based on clinical efficacy were analyzed through clinical drug targets, tumor microenvironments, and tumor stages. Coldness tumors can transform into hotness tumors through chemotherapy, radiotherapy, targeted therapy, and other therapies.

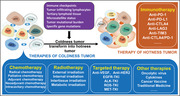

## 1 INTRODUCTION

Cancer morbidity and mortality have continued to rise significantly over the past few years. In 2020, there were 19.29 million new malignant tumor patients were diagnosed worldwide, with 9.96 million cancer‐related deaths. The cancer incidence rate increased significantly compared with 2018. In many countries, malignant tumors have become the leading cause of death.^1^ With the development of society and technological advancements, precision medicine has gradually replaced conventional radiotherapy (RT) and surgical treatment of malignant tumors.^2^, ^3^, ^4^, ^5^, ^6^ Precision cancer therapy is currently the most promising cancer treatment, including targeted therapy and immunotherapy.^7^

Immunotherapy is a therapeutic modality that aims to regulate and eradicate tumors by reactivating and sustaining the antitumor immune cycle, as well as reinstating the body's innate antitumor immune response. It has become the fifth pillar of cancer treatment and mainly includes immune checkpoint inhibitors (ICIs),^8^ adoptive cell transfer therapy,^9^, ^10^, ^11^ tumor‐specific vaccines, cytokines (CKs), and small‐molecule immunological drugs. Of these, ICIs are currently the most widely used drugs in cancer immunotherapy.

Immunotherapy for tumors has been used for over 100 years, but the efficacy of immunotherapy varies even for patients with the same cancer type.^12^, ^13^ In 2009, Camus et al.^14^ introduced the three primary immune features (“hot, variable, and cold”) that are evident in primary colorectal cancers (CRCs). This discovery led to a classification system that is based on the equilibrium between tumor evasion and immune coordination, with a 2‐year recurrence risk of 10, 50, and 80% for the three cancer types, respectively. Variable tumors can further be divided into two distinct patterns: “immune rejection” and “immunosuppressive.”^14^, ^15^ A global consensus immune scoring study validates the prediction of recurrence and survival risk based on these three main tumor subtypes (“hot, variable, and cold”).^16^, ^17^

ICI has been authorized to treat various tumors with demonstrated effectiveness.^18^ Predictive indicators for immunotherapy, which are currently being researched, can be classified into two primary groups. Tumor immune microenvironments (TME) (e.g., tumor‐infiltrating lymphocytes (TILs), tertiary lymphoid structures [TLSs]) fall into the first category.^19^ The second category is related to the molecular characteristics of the tumor cells (microsatellite instability/mismatch repair defects, tumor mutational load, neoantigen load, etc.). “Hot tumors” are characterized by a TME rich in TILs, PD‐L1 overexpression, genomic instability, and preexisting antitumor immune responses.^20^ “Cold tumors” have the opposite characteristics.^21^ Variable tumors are tumors in a variable state between cold and hot ones. It is generally accepted that ICIs alone are more effective against “hot tumors” while having no benefit in treating “cold” tumors or “variable” tumors, which require a combination of other therapies to recruit immune cells to the tumor tissue, that is, to convert a “cold tumor” or “variable” tumor to a “hot” tumor. The conversion of cold tumors and variable tumors to hot tumors is an important option to treat malignant tumors and ought to become a promising and clinically important area of research.

Cancer immunotherapy and ICIs have revolutionized cancer treatment with the development of hot and cold tumors. By taking the immune response of tumor patients as the target of anticancer treatment intervention, combined with chemotherapy, RT, targeted therapy, and other therapeutic methods, anticancer treatment has achieved remarkable and amazing effects, thus completely changing the treatment concept and method in the field of cancer.

The objective of this review is to conduct a comprehensive and in‐depth investigation into the immune mechanism underlying the formation and progression of cancer, examine the interplay between cancer and the immune system of the body, gain a comprehensive understanding of the potential mechanisms and intervention points that influence the emergence of “hot” and “cold” tumors, and offer anticancer concepts and potential strategies that involve combined immunity based on “hot and cold tumors.” We have reason to believe that the comprehensive use of an objective, standardized, and reasonable decision‐making method to guide cancer treatment requires a lot of clinical practice and evidence‐based medical evidence, which requires multidisciplinary collective efforts, but has great clinical value and practical significance.

## 2 CHARACTERISTICS OF COLD AND HOT TUMORS

**FIGURE 1:**
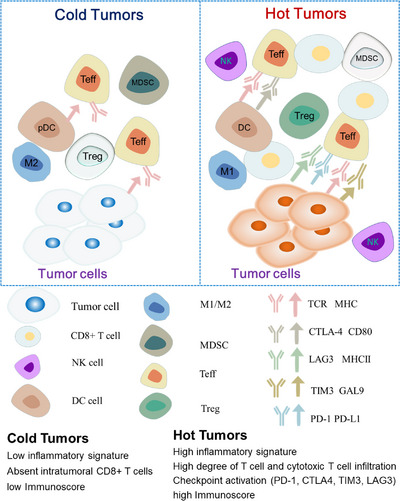
The differences of cold and hot tumors. The main cellular players and molecular interactions that affecting the cold tumor phenotype (left) and the hot tumor phenotype (right). Abbreviations are as follows: NK, natural killer cells; DC, dendritic cells; pDC, plasmacytoid dendritic cells; M1, macrophages of type 1; M2, macrophages of type 2; MDSC, myeloid‐derived suppressor cells; T eff, effector T cells; T reg, regulator T cells; TCR, T cell receptor; MHC, major histocompatibility complex; CTLA‐4, cytotoxic T‐lymphocyte‐associated protein 4; LAG3, lymphocyte‐activationgene‐3; TIM3, T cell immunoglobulin domain and mucin domain‐3; GAL9, galectin‐9; PD‐1, programmed cell death‐1; PD‐L1, programmed cell death‐ligand 1.

**FIGURE 2:**
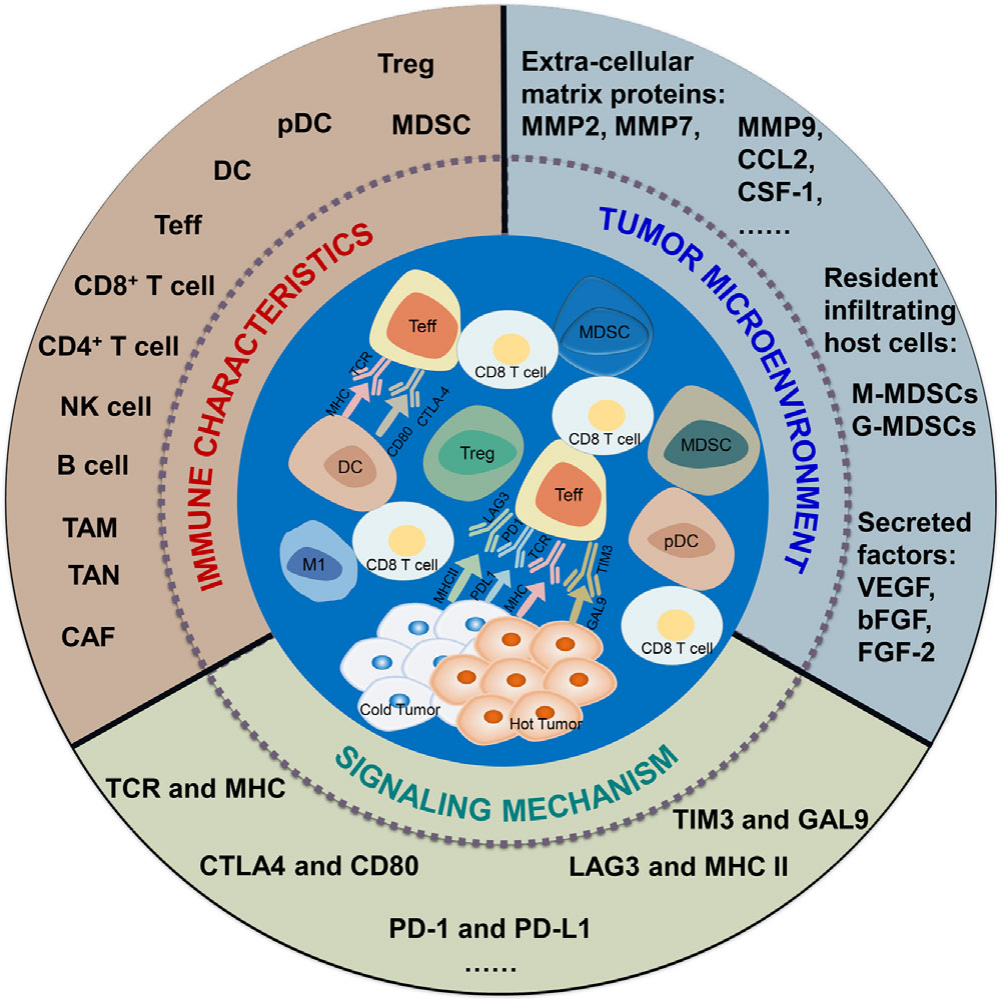
The immune microenvironment of hot and cold tumors. Tumors have many influencing factors in the tumor microenvironment, immune characteristics and signaling mechanisms, and hot and cold tumors present different characteristics, respectively.

The immune microenvironment and immune mechanisms differ between hot and cold tumors (Figure 1), mainly in the degree and type of immune cell infiltration, with different immune characteristics depending on the immune cell activity, and the heterogeneity of TME makes the tumor characteristics vary greatly between individuals (Figure 2).

### 2.1 Tumor microenvironment

The concept of TME was first introduced by Loannnides et al.^19^ in 1993, specifically referring to the local environment of tumor occurrence and development, which mainly includes tumor cells, immune cell stromal cells and their secreted CKs, and extracellular matrix, which is formed through their interactions. The phenomenon of immune infiltration encompasses the presence of all immune cells within the TME, including but not limited to T cells, macrophages, natural killer (NK) cells, bone marrow‐derived suppressor cells (MDSCs), mast cells, neutrophils, B cells, and dendritic cells (DCs), and TLSs resulting from lymphoid neo‐organogenesis involving B cells, T cells, DCs, and lymphatic vessels.^22^, ^23^, ^24^ It is important to note that certain cell types within the TME may exhibit both antitumor and protumor effects. For example, macrophages can differentiate into two simplified categories: M1 macrophages that are associated with acute inflammatory and antitumor activity, and M2 macrophages that are recruited for protumorous chronic inflammation.^25^

### 2.2 Immune characteristics

TME is decisive for tumor heterogeneity, as tumor cells and their surrounding microenvironment are interdependent and influenced by each other. Tumor cells can release extracellular signals and influence TME to produce immune tolerance. Meanwhile, immune cells infiltrating the TME also kill tumor cells, preventing their proliferation and metastasis. The TME comprises two distinct mechanisms of immunosuppression: intrinsic immunosuppression, which may result from genetic alterations within the tumor and involves the activation of multiple oncogenic pathways, leading to the development of cold tumors; and locally adaptive immunosuppression, which gives rise to hot tumors characterized by a high degree of T‐cell infiltration, thereby providing a favorable milieu for effective ICI‐based mono‐ or combination therapy.^20^ To enhance the efficacy of ICIs, it is imperative to devise strategies aimed at modulating the TME and shifting the tumor phenotype from cold to hot.

### 2.3 Signaling mechanism

TMEs with cold or hot tumors are characterized by the presence of sufficient immune cells, immune factors, or other immune factors. The immune microenvironment of cold tumors has fewer immune cells such as T cells and NK cells due to the activation of immunosuppressive pathways of immune checkpoints (ICs) such as PD‐1/PD‐L1 and CTLA‐4, and more immunosuppressive factors like interleukin 10 (IL‐10) and transforming growth factor beta, thus inhibiting the proliferation and activation of immune cells, while IC molecules such as PD‐L1 on the surface of tumor cells also inhibit the activation of immune cells. In contrast, the immune microenvironment of hot tumors is enhanced by the activation of immune activation pathways such as CD28/B7, CD40/CD40L, a higher number of immune cells, mainly including T cells, NK cells, and DCs, fewer immunosuppressive factors and a strong immune response.^26^ The proliferation and activation of immune cells are stimulated by immune activating factors released from tumor cells, while IC molecules such as PD‐L1 on the surface of tumor cells are also attacked and cleared by immune cells.

## 3 FACTORS AFFECTING TUMOR HOTNESS AND COLDNESS

**FIGURE 3:**
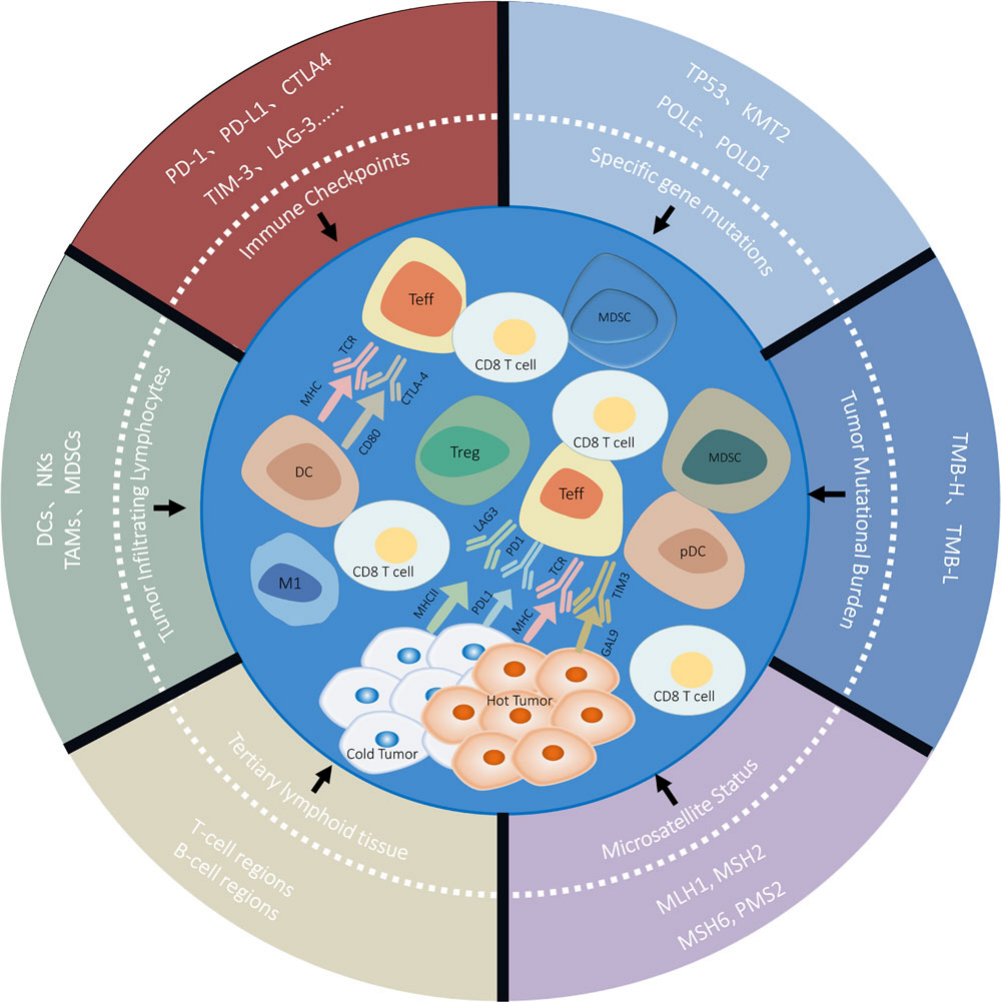
Factors influencing “hot and cold tumors.” “Hot and cold tumors” is a concept in comparison with the clinical efficacy of tumor treatment. Tumors that respond well to treatment are defined as hot tumors, and those that respond poorly are defined as cold tumors. There are many factors affecting tumor efficacy, including immune checkpoints (PD‐1, PD‐L1, CTL4, TIM‐3, LAG‐3), tumor infiltrating lymphocytes (TIL), tertiary lymphoid tissue (TLS), microsatellite status (MSI), tumor mutational burden (TMB), and specific gene mutations (SGM, e.g., TP53, KMT2, POLE, and POLD1). Abbreviations are as follows: TP53, tumor protein p53; KMT2, histone–lysine N‐methyltransferase 2; POLE, polymerase epsilon; POLD1, polymerase delta 1; MLH1, MutL homolog 1; MSH2, MutS homolog 2; MSH6, MutS homolog 6; PMS2, postmeiotic segregation increased 2.

Presently, the terminology “hot” and “cold” is frequently employed to denote tumors that are infiltrated by T cells, those that are inflamed but not infiltrated, and those that are noninflamed. The differentiation between hot and cold tumors is predicated on the status of cytotoxic T cells within the tumors. In addition to the presence of TILs, other attributes, such as the expression of antiprogrammed death ligand 1 (PD‐L1) on immune cells associated with the tumor, potential genomic instability, and preexisting antitumor immune responses, have been identified as hallmarks of hot tumors (Figure 3).

### 3.1 Immune checkpoints

ICs (Table 1)^27^, ^28^, ^29^, ^30^, ^31^, ^32^, ^33^, ^34^, ^35^, ^36^, ^37^, ^38^, ^39^, ^40^, ^41^, ^42^, ^43^, ^44^, ^45^, ^46^, ^47^, ^48^, ^49^, ^50^, ^51^, ^52^, ^53^, ^54^, ^55^, ^56^, ^57^, ^58^, ^59^, ^60^, ^61^, ^62^, ^63^, ^64^, ^65^, ^66^ are inhibitory receptors expressed on T cells or other immune cells, including PD‐1, cytotoxic T‐lymphocyte‐associated protein‐4 (CTLA4), T cell immunoglobulin and mucin‐domain containing‐3 (TIM‐3), and lymphocyte activation gene‐3 (LAG‐3). Ligand molecules of IC have been found to be widely expressed in various types of tumor cells and may be involved in tumor immune escape through different mechanisms of action.

**TABLE 1:** IC‐related drugs and their indications.

Target	Drugs	Indications	References
Anti‐PD‐1	Pembrolizumab	Non‐small cell lung cancer; melanoma; head and neck squamous cell cancer; classical Hodgkin lymphoma; primary mediastinal large B‐cell lymphoma; urothelial carcinoma; microsatellite instability‐high or mismatch repair deficient cancer; gastric cancer; esophageal cancer; cervical cancer; hepatocellular carcinoma; Merkel cell carcinoma; renal cell carcinoma; endometrial carcinoma; tumor mutational burden‐high cancer; cutaneous squamous cell carcinoma; triple‐negative breast cancer	^27^, ^28^
	Nivolumab	Melanoma; non‐small cell lung cancer; malignant pleural mesothelioma; renal cell carcinoma; classical Hodgkin lymphoma; squamous cell carcinoma of the head and neck; urothelial carcinoma; microsatellite instability‐high or mismatch repair deficient metastatic colorectal cancer; hepatocellular carcinoma; esophageal cancer; gastric cancer, gastroesophageal junction cancer, esophageal adenocarcinoma	^29^, ^30^, ^31^, ^32^
	Dostarlimab	Mismatch repair deficient recurrent or advanced endometrial cancer	^33^, ^34^
	Toripalimab	Melanoma; urothelial carcinoma; esophageal cancer; nasopharyngeal carcinoma	^35^, ^36^
	Tislelizumab	Classical Hodgkin lymphoma; urothelial carcinoma; non‐small cell lung cancer; hepatocellular carcinoma; microsatellite instability‐high or mismatch repair deficient cancer; esophageal cancer; nasopharyngeal carcinoma	^37^, ^38^
	Camrelizumab	Classical Hodgkin lymphoma; hepatocellular carcinoma; esophageal cancer; nasopharyngeal carcinoma; non‐small cell lung cancer	^39^, ^40^
	Sintilimab	Classical Hodgkin lymphoma; non‐small cell lung cancer; hepatocellular carcinoma; esophageal squamous cancer; gastric cancer	^41^, ^42^
	Penpulimab	Classical Hodgkin lymphoma	^43^, ^44^
	Zimberelimab	Classical Hodgkin lymphoma	^45^, ^46^
	Serplulimab	Microsatellite instability‐high or mismatch repair deficient cancer; non‐small cell lung cancer	^47^, ^48^, ^49^
	Pucotenlimab	Microsatellite instability‐high or mismatch repair deficient cancer; melanoma	^50^, ^51^
Anti‐ PD‐L1	Atezolizumab	Non‐small cell lung cancer; small cell lung cancer; hepatocellular carcinoma; melanoma; alveolar soft part sarcoma	^52^, ^53^
	Durvalumab	Non‐small cell lung cancer; small cell lung cancer; biliary tract cancers; hepatocellular carcinoma	^54^, ^55^
	Envafolimab	microsatellite instability‐high or mismatch repair deficient cancer	^56^, ^57^
	Sugemalimab	Non‐small cell lung cancer	^58^, ^59^, ^60^
	Adebrelimab	Small cell lung cancer	^61^, ^62^
Anti‐CTLA4	Ipilimumab	Unresectable or metastatic melanoma; adjuvant treatment of melanoma; advanced renal cell carcinoma; microsatellite instability‐high or mismatch repair deficient metastatic colorectal cancer; hepatocellular carcinoma; metastatic non‐small cell lung cancer; malignant pleural mesothelioma; esophageal cancer	^63^, ^64^
Anti‐CTLA4/PD‐1	Cadonilimab	Cervical cancer	^65^, ^66^

**FIGURE 4:**
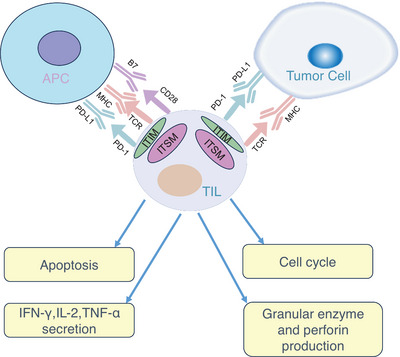
PD‐1 and PD‐L1 interactions affect TIL function in the tumor microenvironment. In the tumor microenvironment, PD‐L1, which can bind PD‐1, is aberrantly highly expressed on tumor cells and APC. Activation of PD‐1/PD‐L1 signaling induces phosphorylation of tyrosine residues in the PD‐1 cytoplasmic ITIM and ITSM structural domains and, by inducing apoptosis, inhibiting the production of granzyme and perforin, reducing the secretion of IFN‐γ, IL‐2, and TNF‐α reduces the antitumor activity of TILs and arrests the cell cycle. Abbreviations are as follows: TCR, T cell receptor; MHC, major histocompatibility complex; APC, antigen‐presenting cell; ITIM, immune receptor tyrosine‐based inhibitory motif; ITSM, immune receptor tyrosine‐based switch motif.

PD‐1 is a cell surface receptor that binds to PD‐L1 ligand in the TME to activate downstream signaling pathways to inhibit T cell activation, thereby causing tumor‐specific T cell failure and apoptosis.^67^, ^68^ PD‐1 is abundantly expressed in various cancers, resulting in the immune escape of tumor cells and promoting malignant tumor progression.^69^, ^70^, ^71^ Combined action of PD‐1 and PD‐L1 suppresses host antitumor immunity, leading to tumor immune escape (Figure 4).

PD‐L1 is the most relevant predictive marker for the treatment efficacy of ICIs.^72^ The main evaluation indicators include TPS (tumor cell proportion score), CPS (combined positive score), and IPS (immune cell proportion score). In contrast, the TPS score is less well established, while the CPS is a more stable and reproducible method of scoring PD‐L1.^73^ A study finds the objective response to pembrolizumab in gastric cancer patients significantly correlates with CPS but not TPS.^74^ Therefore, CPS perhaps is a more accurate index for PD‐L1 evaluation than TPS. According to the different CPS scores, tumors can be classified into three PD‐L1 expression‐based subtypes: CPS < 1 is considered no expression, which is equivalent to “cold” tumors; 1 ≤ CPS < 50 is considered low expression, which is equivalent to “variable” tumors; when CPS ≥50, the tumor is considered to be highly expressed, corresponding to a “hot” tumor.^75^, ^76^

CTLA‐4 is predominantly expressed on activated T cells and Tregs, functioning as a negative regulator for T cell activation. The use of CTLA‐4 inhibitors plays a significant role in T cell initiation and can facilitate T cell trafficking to “cold” tumors. TIM‐3 primarily serves to inhibit the activation and proliferation of T cells, thereby enabling immune evasion by tumor cells. Overexpression of TIM‐3 is linked to T cell dysfunction and exhaustion. LAG‐3, a cell surface protein of the immunoglobulin superfamily, acts similarly to CTLA4 and PD‐1 and negatively regulates T cell activation.

### 3.2 Tumor‐infiltrating lymphocytes

TILs are immune cells isolated from tumor tissue and contain both positive regulatory immune cells, such as DCs, CD8+ T cells, and NK cells,^77^, ^78^ and negative regulatory immune cells, such as tumor‐associated macrophages (TAMs), regulatory T cells, myeloid‐derived suppressor cells (MDSCs), tumor‐associated neutrophils (TANs), and son.^79^, ^80^ TILs are evaluated by HE staining, immunohistochemistry, multiplex immunofluorescence, flow cytometry, and so on.^81^, ^82^, ^83^

In a study of 228 immunotherapy patients with mNSCLC, the patients were classified into four types based on levels of CD8+ TILs and PD‐L1, and significant differences in response to PD‐1/PD‐L1 monoclonal antibodies were found among the different immunotypes. For the patients with high expression of PD‐L1, ORR and PFS were significantly better in type I patients with high expression of TILs than in type III patients with low expression of TILs.^84^ Another study of patients with stage II or stage III CRC found that patients with low TIL had poor disease‐free survival (DFS) and those with high TIL had a better prognosis, regardless of whether they were dMMR or pMMR.^85^

### 3.3 Tertiary lymphoid tissue

TLS, the third type of lymphoid tissue distinct from primary lymphoid tissue (thymus and bone marrow) and secondary lymphoid tissue (spleen, lymph nodes, tonsils, etc.), consists of multiple immune cells, including T‐cell and B‐cell regions,^86^, ^87^ that promote immune cell infiltration into solid tumors.^88^ TLS has been shown to predict the efficacy of ICIs in multiple tumor types and is independent of PD‐L1 status.^89^ The first report of TLS appeared in a study in which samples from NSCLC patients were tested, and TLS (clusters of mature DCs and T cells) were found to be present at the infiltrative margins of NSCLC tumors at all stages and were described as tumor‐induced bronchial‐associated lymphoid tissue.^90^

Many clinical studies have shown that TLS cell composition, density, and site correlate with improved clinical prognosis or benefit from immunotherapy in lung cancer,^91^, ^92^ ovarian cancer,^93^ melanoma,^94^, ^95^ CRC,^96^ head and neck cancer,^97^ and oral squamous cell carcinoma.^98^ However, it has also been shown that TLS is associated with poorer tumor prognosis, for example, in hepatocellular carcinoma; additionally, TLS is associated with tumor progression, poorer associated clinicopathological features, and poor prognosis.^99^, ^100^ Different stages of TLS differentiation within the tumor may play different roles in tumor immunity, with patients containing mature TLS presenting lower recurrence rates compared to those containing early TLS in CRC^96^, ^101^ and liver cancer.^100^

### 3.4 Microsatellite status

Microsatellite (MS), also known as short series repeated sequence, is a simple repetitive sequence of less than 10 nucleotides in the DNA genome.^102^ Mismatch Repair (MMR) genes include MLH1, MSH2, MSH6, and PMS2.^103^, ^104^ Microsatellite status (MSI) can be caused by MMR‐Foricient (D‐MMR) genetic mutations or expression abnormalities.^105^, ^106^ According to the number of mutation sites, MSI can be divided into MSI‐H, MSI‐L, and MSS. Among them, MSI‐L and MSS are often classified as a single category because of their highly similar clinical characteristics, which are equivalent to “cold tumors” or “variable tumors.”^107^

In cancer treatment, dMMR and MSI‐H are considered as “hot tumors” because of their sensitivity to ICIS treatment.^108^, ^109^ The advanced MSI‐H solid tumors often respond well to immunotherapy. Some studies have shown that the benefit of ICI treatment for tumor patients is closely related to the levels of MSI and has nothing to do with specific cancer types.^110^ Patients with dMMR/MSI‐H CRC can get better disease control through ICI treatment.^111^, ^112^ One study confirmed that the median follow‐up times for MSI‐H CRC and MSI‐H non‐CRC cancers were 7.4 months and 4.5 months, respectively, with ORRs of 26.2% and 42.9% and DCRs of 50.8% and 66.7%, both achieving safe and efficient treatment outcomes.^113^ In studies of pembrolizumab for the treatment of non‐CRC patients with MSI‐H/dMMR, the ORR was 48% and the DCR 66% in patients with endometrial cancer^114^ and 45.8% in patients with gastric cancer.^115^ With a median PFS of 11.0 months, pembrolizumab showed potent antitumor activity, producing a durable response. The incidence of MSI‐H varied by cancer and was described in only about 5% of cancer patients, being more common in CRC, gastric cancers, and endometrial adenocarcinomas.

### 3.5 Tumor mutational burden

Tumor mutational burden (TMB) is the number of nonsynonymous mutations occurring in the tumor genome, reflecting the degree of genomic variation in the tumor.^116^ TMB‐H (high) tumors have the potential to acquire more de novo antigens, which can improve tumor immunogenicity and response to ICI,^117^ and TMB has become an important indicator for immunotherapy dosing. One study found a correlation between immunotherapy and TMB, with high TMB values predicting better treatment outcomes.^118^, ^119^

In June 2020, the United States Food and Drug Administration (US FDA)‐approved pembrolizumab for the treatment of unresectable or metastatic solid tumors with TMB‐H.^120^ According to some studies, TMB predicts the efficacy of ICI in patients with malignant melanoma and NSCLC; patients with TMB ≥ 20 mutations/mb responded better to immunotherapy and had a longer DFS.^121^ Pembrolizumab had better efficacy in patients with TMB‐H compared with patients with TMB‐L (low) (ORR, 30.3 vs. 6.7%). Another analysis of pan‐cancer study of 1600 patients also showed that TMB‐H was associated with higher response rates and longer OS with ICIs.^122^ Therefore, the US FDA‐approved pembrolizumab for monotherapy in TMB‐H and unresectable or metastatic solid tumors with disease progression after prior therapy, regardless of cancer type.^123^

### 3.6 Specific gene mutations

The TP53 gene is a carcinogenic suppression gene that maintains genome stability on the 17th chromosome and prevents genetic mutation.^124^, ^125^ TP53 mutation status may be a positive or negative predictor of response to tumor immunotherapy (e.g., a positive predictor in breast and lung adenocarcinomas and a negative predictor in gastric, colon, and head and neck squamous cell carcinomas).^126^ TP53 mutations increased TAMs in the TME of lung, ovarian, pancreatic, and skin cancers,^127^ involving multiple complex signaling pathways, including regulation of gene expression, protein stability, protein interactions, and posttranscriptional modifications,^19^ causing changes in the secretion of chemokines and CKs, which in turn alter the immune microenvironment.

Cell adhesion molecules (CAMs), a class of proteins that mediate adhesion between cells and between cells and external structures, play an important role in tumor development and metastasis. Tumor endothelial cells (ECs) promote the adhesion of tumor cells to vascular ECs and inhibit the infiltration of T cells into the tumor bed by modulating CAMs (ICAM‐1 and VCAM‐1). Hot tumors are characterized by a high infiltration of various infiltrating lymphocytes (TILs), whereas cold tumors have fewer infiltrating lymphocytes and often show abnormal expression of CAMs on tumor‐associated vessels, thus preventing immune cells from entering the tumor. Clinical studies have demonstrated that upregulation of ICAM‐1 in the TME is associated with a good prognosis for patients with various cancers, suggesting enhanced immune surveillance of cancer.^128^, ^129^, ^130^, ^131^

In addition, several other studies have demonstrated that some specific gene mutations (e.g., KMT2,^132^ POLE, and POLD1^133^) can be used as independent predictors of survival benefit from treatment with pan‐tumoral ICIs.

## 4 TREATMENT STRATEGIES FOR HOT AND COLD TUMORS

**FIGURE 5:**
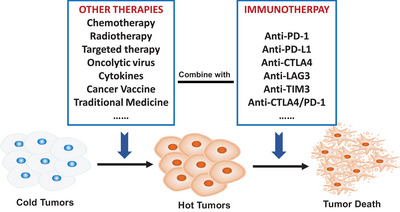
Therapeutic strategies for cold and hot tumors. For hot tumors with high PD‐L1 expression and MSI‐H, single immunotherapy can show amazing efficacy, while for cold tumors with low PD‐L1 expression and MSI‐L, single immunotherapy cannot achieve satisfactory efficacy. How to convert a cold tumor into a hot tumor is crucial. Combined immunotherapy strategy is an important method for cold tumor treatment, including immunotherapies combined with chemotherapy, targeted therapy, radiotherapy, double immunotherapy, oncolytic virus, cancer vaccine, cytokines, traditional medicine, intestinal flora, and so on, are all clinically available combination methods.

In both cold and hot tumors, the focus of treatment is on activating the immune system and enhancing the ability of the immune system to kill tumor cells. The relevance of tumor classification criteria and the effectiveness of immunotherapy can guide the selection of different immunotherapy strategies for different types of tumors in clinical applications (Figure 5).

### 4.1 Hot tumors treatment strategies

“Hot” tumors provide fertile ground for immunotherapy through high levels of T‐cell infiltration, and ICIs can block the PD‐1/PD‐L1 pathway or CTLA‐4, normalizing the immune system and inhibiting tumor growth. The “hot tumor” treatment focuses on strengthening the immune system against the tumor and increasing the activity of the immune cells.

#### 4.1.1 Anti‐PD‐1 monotherapy

In 2017, the US FDA‐approved nivolumab for the treatment of MSI‐H/dMMR mCRC patients who had progressed after prior chemotherapy.^112^ Pembrolizumab is available as a first‐line treatment option for NSCLC patients without EGFR or ALK mutations and with PD‐L1 TPS ≥ 50%.^134^ Cemiplimab was approved as a single‐agent first‐line treatment for patients with locally advanced or metastatic NSCLC with ≥50% PD‐L1 expression and no EGFR, ALK, or ROS1 mutations and who are not candidates for surgical resection or RT.^135^ Sintilimab was approved as a single‐agent treatment for EGFR‐ and ALK‐negative patients with PD‐L1 TPS ≥ 50.^136^ Toripalimab was effective in treating advanced UC patients with PD‐L1‐positive TMB‐H.^137^ Camrelizumab monotherapy showed beneficial antitumor activity in patients with PD‐L1‐positive pulmonary sarcomatoid carcinoma.^138^ Tislelizumab was used to treat adult patients with unresectable or metastatic MSI‐H/dMMR in advanced solid tumors.^139^

#### 4.1.2 Anti‐PD‐L1 monotherapy

Atezolizumab was approved in China as first‐line monotherapy for metastatic NSCLC with high PD‐L1 expression and no EGFR/ALK gene mutations.^140^ A phase I/II clinical study of duravulumab for the first‐line treatment of NSCLC patients provided an ORR of 28.6% in PD‐L1‐positive patients.^141^ In patients with MSI‐H/dMMR mCRC, avelumab (avelumab) outperformed second‐line standard therapy in terms of PFS.^142^ Envafolimab has a better efficacy and safety profile in the treatment of patients with dMMR/MSI‐H tumors.^56^

#### 4.1.3 Anti‐PD‐1/CTLA4 bi‐specific antibody

There is enhanced antitumor efficacy of cadonilimab, a drug that blocks the ligands to bind on PD‐1 and CTLA‐4^65^ with a 43.8% ORR and a median PFS of 6.34 months in PD‐L1‐positive cervical cancer patients (CPS ≥ 1).^143^

### 4.2 Cold and variable tumors treatment strategies

Single immunotherapy can achieve good results in “hot” tumors, but few patients meet the treatment criteria, and the treatment costs are high. Moreover, the intricate nature of immunomodulatory signaling pathways implies that patients who exhibit initial sensitivity to ICI therapy may eventually experience resistance or relapse.^144^ Consequently, it is imperative to explore methods that can enhance ICI sensitivity. The integration of targeted therapy, chemotherapy, and RT can potentially transform “cold” or “variable” tumors into “hot” ones, thereby augmenting the effectiveness of immunotherapy. Furthermore, novel and personalized immunotherapy approaches should be investigated to improve patients’ sensitivity and response rates to activated antitumor immune responses.^145^

#### 4.2.1 Combination of chemotherapy with PD‑1/PD‑L1 inhibitors

Chemotherapy is a commonly used treatment modality for cancer by targeting DNA replication and disrupting cellular metabolism.^146^ Certain cytotoxic chemotherapeutic agents, such as anthracyclines and oxaliplatin, have been shown to induce immunogenic cell death (ICD), stimulate antitumor immune responses,^147^ or directly eliminate immunosuppressive cells, and enhance the function of effector cells.^148^, ^149^ Appropriate combinations of chemotherapeutic agents and ICIs can enhance efficacy and produce a more durable antitumor response.^150^, ^151^

In 2016, the US FDA approved the use of pembrolizumab in combination with pemetrexed/carboplatin for the treatment of metastatic nonsquamous NSCLC, which effectively improved symptoms and increased PFS.^152^, ^153^ PD‐1 monoclonal antibody in combination with GEM (gemcitabine) has demonstrated efficacy in treating of hepatic metastases from pancreatic ductal adenocarcinoma enhanced the immune response and significantly prolonged OS in mice model.^154^ One study evaluated changes in IC ligands and receptors on the cell surface of esophageal adenocarcinoma (OAC) before and after chemotherapy demonstrated that chemotherapy upregulated PD‐L1 and TIM‐3 on the surface of OAC cells to enhance the stem cell‐like phenotype and promote the conversion of “cold” to “hot” tumors, making them more sensitive to ICI treatment.^155^

#### 4.2.2 Combination of molecular targeting drugs with PD‑1/PD‑L1 inhibitors

Vascular endothelial growth factor (VEGF) regulates the growth of vascular ECs, causing immunosuppression.^156^ Several studies have shown that blocking VEGF‐related pathways enhances the immune response, and the combined application of PD‐L1 monoclonal antibodies and VEGF inhibitors has shown good synergistic effects in clinical studies and is promising for the treatment of a variety of tumors.^157^ PD‐L1 inhibitors in combination with VEGFR2 inhibitors can significantly downregulate PD‐1 and PD‐L1 expression levels, increase TILs, decrease Treg and MDSCs, and inhibit tumor growth.^158^, ^159^ Bevacizumab (bevacizumab), an antiangiogenic drug, increased T cell reversal of immunosuppressive infiltration and improved the antitumor activity of the PD‐L1 monoclonal antibody.^160^ Atezolizumab in combination with Bevacizumab improved PFS and OS in patients with mNSCLC, and the combination significantly improved Atezolizumab sensitivity.^161^

EGFR is a transmembrane tyrosine kinase receptor involved in tumor cell proliferation, invasion, and metastatic angiogenesis.^162^ T‐cell apoptosis is reduced and IFN‐ production is increased by EGFR inhibitors (EGFR‐TKI).^163^ Erlotinib, an EGFR‐TKI, reduces CD4 effector regulatory T‐cell infiltration in TME and is used in combination with PD‐1 monoclonal antibody to treat NSCLC, improving immunotherapy efficacy.^164^ In EGFR/ALK‐positive and ALK‐rearranged patients, the addition of bevacizumab improved the clinical outcome of ICI.^165^

Lenvatinib is a drug that helps transform TME and improve the anticancer effect of immune cells^166^ by inhibiting both VEGFR and FGFR targets.^167^ In September 2019, Lenvatinib in combination with pembrolizumab (the “cola combination”) received US FDA approval for patients with advanced endometrial cancer who have disease progression after systemic therapy that is not suitable for surgery or RT and who do not have MSI‐H or dMMR.^168^ In a phase IB single‐arm study of first‐line treatment of advanced hepatocellular carcinoma, the “cola combination” improved tumor activity with an ORR of 76.7%, a CR rate of 10%, and a significant prolongation of patient OS.^169^ In another phase II study, the ORR of “cola combination” was 21.4% and the mOS was 13.9 months in patients with inoperable stages III–IV melanoma who had progressed to resistance to previous immunotherapy, demonstrating good efficacy and a manageable safety profile.^170^ Based on the positive results of these clinical studies, the “cola combination” has become a classic combination of immunotherapy and targeted therapy.

#### 4.2.3 Dual IC blockade or costimulatory molecule agonist plus α‑PD‑1/PD‑L1

Dual immunotherapy, referring to the combination of immunotherapies targeting two different mechanisms or targets of action, is gradually gaining acceptance among clinicians and cancer patients as clinical studies on dual immunotherapy are conducted and the benefits of efficacy have been realized.^171^, ^172^, ^173^ Nivolumab and Ipilimumab are PD‐1 antibody and CTLA‐4 antibody: one reduces excessive contact between T cells and cancer cells to prevent T cells from being misled by cancer cells,^174^ and the other signals antigen‐presenting cells to activate T cells^174^, ^175^ for cancer cell killing. The combination of PD‐1 and CTLA‐4 inhibitors is mechanistically synergistic and complementary,^176^, ^177^ and Nivolumab and Ipilimumab are the only dual immunotherapies currently approved by the US FDA,^178^, ^179^ demonstrating better antitumor effects than single immunotherapies, especially in “cold tumors.”^171^

There are also ICs that are still in clinical trials and have not yet been approved by the US FDA or NMPA. Lymphocyte‐activation gene 3 (LAG‐3) negatively regulates T‐cell effects by directly inhibiting helper T cells (Th) via MHCII.^180^, ^181^ In the EG7 lymphoma model, the combination of anti‐LAG3 and PD‐1 monoclonal antibodies had a 100% tumor clearance effect, whereas treatment with PD‐1 monoclonal antibodies alone resulted in a tumor clearance rate of only 50%.^182^ Targeted inhibition of LAG3 and PD‐1 also showed significant tumor regression in the B16‐F10 recurrent melanoma model. Tim‐3 (T cell immunoglobulin domain and mucin domain‐3, TIM‐3) on effector T cells interacts with Galectin‐9 on tumor cells to induce T cell apoptosis and suppress immune responses.^183^ TIL is a severely depleted phenotype that fails to proliferate to produce IL‐2, TNF, and IFN‐. It is TIM‐3 and PD‐1 positive. The combination of a TIM‐3 inhibitor and a PD‐1 monoclonal antibody can upregulate TIL expression to effectively control tumor growth in lung cancer mice, whereas the use of PD‐1 monoclonal antibodies alone led to resistance, promoting tumor progression.^184^, ^185^

#### 4.2.4 Combination of RT with PD‑1/PD‑L1 inhibitors

Radiotherapy (RT) can enhance the efficacy of PD‐1 inhibitors by promoting T‐cell infiltration, increasing the number of TILs, inducing ICD, and enhancing the antitumor immune response.^186^, ^187^, ^188^ On the one hand, it can induce damage‐associated molecular patterns (DAMPs) and CKs (especially IFN‐I) associated with ICD to recruit immune cells and promote DC function; on the other hand, DCs can capture their released tumor antigens and present them to T cells^189^, ^190^ as a way to stimulate systemic antitumor immunity.

Most radioimmunotherapy regimens are based on stereotactic body radiation therapy (SBRT), which can deliver ablative doses of radiation by means of image guidance and intensity modulation.^191^, ^192^, ^193^ SBRT combined with durvalumab was superior to durvalumab alone in early‐stage NSCLC, and the major pathological response rate was significantly higher than in the durvalumab group.^194^ Clinical studies have demonstrated that anti‐PD‐1 therapy significantly improves OS in patients with melanoma brain metastases treated with stereotactic radiotherapy.^195^

#### 4.2.5 Combination of oncolytic virus with PD‑1/PD‑L1 inhibitors

Oncolytic virus (OV) therapy elicits ICD and immune responses via the release of pathogen‐associated molecular patterns and DAMPs,^196^, ^197^ resulting in the destruction of cancer cells by targeting the tumor vascular system and inducing immunity,^198^, ^199^ increasing the sensitivity of tumor cells to immunotherapy (“warming up” the tumor), and enhancing the therapeutic effect.

Talimogene laherparepvec, the first US FDA‐approved oncolytic therapy, is a herpes simplex virus that expresses granulocyte‐macrophage colony‐stimulating factor.^200^ In Talimogene combined with pembrolizumab in 21 patients with advanced melanoma, lysing virus therapy was found to improve immune efficacy by altering TME.^201^ A phase 1 clinical trial of a herpes simplex virus type 1‐based lysozyme virus (HSV‐1‐G207) for the treatment of high‐grade gliomas in children showed that intratumoral injection of the lysozyme virus alleviated recurrent or progressive high‐grade gliomas in children, transforming “cold” tumors to “hot” tumors.^148^ Poxvirus is a highly immunogenic vector for lytic immunotherapy,^202^, ^203^ and some studies have reported that poxvirus attracts effector T cells in mouse models of colorectal and ovarian cancer.^204^, ^205^ mJX‐594 (JX), a lysing cowpox virus, was found to be effective in restoring peritoneal immunity by Lee et al.^206^ This virus promoted immune cell infiltration, inhibited peritoneal metastasis of colon cancer cells, and exerted stronger antitumor effects through combined immunotherapy.^206^ t‐VEC, an OV for the treatment of malignant melanoma, provides a new strategy for melanoma treatment by upregulating the expression of inflammatory molecules, including PD‐L1, and enhancing immune efficacy.^207^ Rotavirus vaccines have immunostimulatory and antitumor effects,^208^ and when used in oncology, it can overcome resistance to PD‐L1 inhibitors and have synergistic effects with them. Rotaviruses have been used clinically and can be used for clinical sensitization of anti‐PD‐1/PD‐L1 therapy.^209^

#### 4.2.6 Combination of cancer vaccine with PD‑1/PD‑L1 inhibitors

Tumor vaccines control or eliminate tumors by increasing immunity and activating the patient's immune system,^210^, ^211^ The DNA vaccine contains the coding sequences of multiple neoantigens and is a universal and personalized cancer treatment.^212^, ^213^

Studies have shown that DNA vaccines combined with anti‐PD‐1 treatment significantly controlled tumor growth and achieved a 25% cure rate in MC38 colon cancer cell line‐inoculated hormonal mice, demonstrating the synergistic effect of tumor vaccines and ICIs.^214^ The lmdd‐MPFG vaccine promotes the TAMs NF‐κB pathway and autophagy pathway in hepatocellular carcinoma cells by activating PD‐L1 expression, restoring T‐cell responses to PD‐1 inhibitors, and enabling local T‐cell resensitization to anti‐PD‐1 immunotherapy in tumors.^215^ A study investigated Nivolumab in combination with a peptide vaccine as adjuvant therapy for patients with stages III and IV resected melanoma, demonstrating the combination significantly increased CD8+ T cell levels and enhanced treatment efficacy.^216^, ^217^

HER2Δ16 commands an important oncogenic signaling pathway; however, endogenous HER2Δ16+ breast cancers do not respond to single‐dose treatment of anti‐PD‐1. One mouse model study found that the use of anti‐PD‐1 in combination with Ad‐HER2Δ16‐KI improved survival, with approximately 30% of mice showing complete tumor regression as well as long‐term tumor‐free survival, and HER2Δ16 vaccine effectively induced HER2‐specific T cells in TME, enhancing the therapeutic effect.^218^ One study created a liposomal nanoparticle encapsulated with TNF‐α that penetrated into and remained in tumor tissue for an extended period of time, promoting ICD, inducing the release of tumor‐specific antigens, promoting T‐cell infiltration, converting tumor cells into endogenous vaccines, therefore significantly improving antitumor immunity of PD‐1/PD‐L1 antibodies.^219^ cMB305 (DC vaccine) in combination with atezolizumab activated antitumor‐specific immune responses in some patients with synovial sarcoma, demonstrating enhanced effect of CMB305 vaccine against PD‐L1 immunotherapy.^220^

#### 4.2.7 Combination of CK treatment with PD‑1/PD‑L1 inhibitors

CKs are low‐molecular‐weight soluble proteins induced by immunogens, mitogens, or other stimulants to be produced by a variety of cells. They are an essential part of the TMB, having an important role in tumor pathogenesis.^221^

ALT‐803 (IL‐15 superagonist) in combination with ICI for refractory solid tumors enhances immune efficacy.^222^, ^223^, ^224^, ^225^ Phase Ib clinical trials of ALT‐803 in combination with nivolumab for NSCLC resulted in reactivation of antitumor activity.^226^ In PD‐L1‐negative (<1%) nonsquamous NSCLC patients, only 9% achieved clinical efficacy with nivolumab alone, whereas the combination of ALT‐803 and nivolumab demonstrated a clinical efficacy of 30%, indicating that the utilization of IL‐2Rβγ agonists may serve as a potential solution to overcome resistance to ICI therapy. The activation of proinflammatory CKs such as IFN‐γ systemically stimulates an effector immune response, thereby facilitating the conversion of “cold” tumors to “hot” tumors.^227^

Additional research has demonstrated that L19‐IL‐2 treatment significantly enhances the infiltration of immune cells (e.g., NK cells, T cells) into tumor tissue in mice compared to IL‐2 treatment and exhibits significantly stronger tumor growth inhibition, with L19‐IL‐2 showing good antitumor activity as a single or combination therapy for metastatic solid tumors in phase I clinical studies.^228^, ^229^

#### 4.2.8 Combination of traditional Chinese medicine with PD‑1/PD‑L1 inhibitors

With modern traditional Chinese medicine (TCM) research at the molecular level, study attempts have been made to combine TCM to the effects on the PD‐1/PD‐L1 signaling pathway.^230^, ^231^, ^232^ Recent studies have found that many TCM extracts/components such as paeoniflorin,^233^ berberine,^234^ cordycepin,^235^ atractylenolide I,^236^ can regulate lymphocyte PD‐1/PD‐L1 expression, modulate tumor immunity, and enhance antitumor effects by increasing the proliferation and killing effect of T lymphocytes such as CD4+.

There are also variable Chinese herbal compounds found to act on cells or CKs in the TMB to remodel the TMB and enhance the body's antitumor immune response. Zhong et al.^237^ administered Guilu Erxian Jiao Decoction gavage to tumor‐bearing mice inoculated with the H22 hepatocellular carcinoma cell line, to find that such gavage inhibit tumor growth by reducing apoptosis of T‐lymphocytes through inhibition of PD‐1 expression. Ge Gen Scutellaria Tang has an overall regulatory effect on immune cells, inhibiting tumor progression by increasing the number of CD8+ T cells and NK cells.^238^

#### 4.2.9 Combination of intestinal microflora with PD‐1/PD‐L1 inhibitors

Several studies have also confirmed that intestinal flora can influence the efficacy of cancer immunotherapy.^239^, ^240^ For example, Bifidobacterium, Bacillus faecalis, Ackermannia, and Bacteroides fragilis, can enhance the function of DCs and T cells and contribute to a better immunotherapeutic response.^241^, ^242^ Oral administration of Bifidobacterium bifidum significantly reduced the growth rate of melanoma, promoted DC maturation and IFN‐γ production, and enhanced the antitumor effects of PD‐1 inhibitors, according to one study.^243^

### 4.3 Research prospects and application perspectives for hot and cold tumors

With the extensive research and therapeutic applications of the concept of “hot and cold” tumors, the future treatment of malignant tumors will be based on gene expression, immunophenotype, and molecular typing of patients, alas gradually downplaying the importance of tumor location and pathological types, therefore ushering in a new conceptual and fundamental change in cancer treatment regime. “Hot tumors“ are more likely to occur in the bladder, head and neck, kidney, liver, melanoma, and non‐small cell lung cancers, while ”cold tumors“ are more likely in gliomas, ovarian, prostate, and pancreatic cancers.^244^ Further studies of ”hot and cold” tumors are still needed, and its conceptual application in the field of tumor therapy is promising.

**FIGURE 6:**
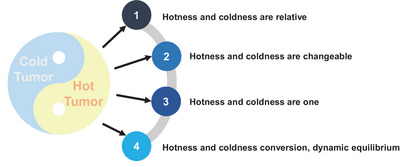
Holistic and yin‐yang properties of tumors. The “hot and cold tumor” is consistent with the “Yin and Yang” attribute in traditional Chinese medicine theory, which believes that “Yin and Yang” are two corresponding attributes of the human body, which are mutually used and dynamically balanced. “Tumor” also has Yin and Yang attributes, “hot and cold tumor” is relative, but also mutual root, can be transformed, can be normalized, dynamic balance.

The “hot and cold” characteristics of tumors are similar to the “yin and yang” properties of TCM.^245^, ^246^ Just as “yin” and “yang” (Figure 6) are mutually rooted and transformed into each other, so can “hot” and “cold” tumors. According to Chinese medicine, the causative factors of malignant tumors, such as phlegm, stasis, and toxicity, are yin‐evil glued to each other and therefore called “yin in the body,” while the active growth and proliferation of tumors, invasion, metastasis, and other malignant behaviors are “yang in the body.”^247^ Interestingly, TCM's understanding of the “yin” and “yang” properties of malignant tumors, analysis of the etiology and pathogenesis, as well as the differentiation and classification of malignant tumors have all reflected the “hot and cold” concept of malignant tumors. A lot of clinical and basic studies have been conducted on the treatment of “hot and cold” tumors in Chinese medicine, and some active ingredients and herbal compounds have been proven to be able to adjust “hotness and coldness” of the immune function of tumor patients via affecting immune organs, immune cells, and immune molecules.^248^ By analyzing the “yin” and “yang” of “hot and cold” tumors, TCM treats “cold” tumors by “warming the yang and supporting the righteousness,” converting the tumor from being “cold” to “hot,” to activate the positive immunity of “cold tumors” to achieve effective clinical treatment of malignant tumors.

The efficient application of immunotherapy in the era of precision medicine requires clear information on tumor gene mutation profiles and immunophenotypes. Various tumor immune markers are used for patient selection of ICI treatment, also guiding us in designing studies to combine basic research and clinical scenarios to transform ICI insensitive cancers into sensitive ones. This strategy, combined with developments in precision medicine and the use of next‐generation sequencing in molecular profiling of cancers, will take us into the era of personalized cancer immunotherapy, where multiple therapeutic approaches are combined to achieve durable remission in hard‐to‐treat cancers.

## 5 SUMMARY AND PROSPECT OF THE FUTURE

“Cold” and “hot” have attracted widespread interest because of their important impacts on cancer immunotherapy.^249^, ^250^, ^251^ The “hot” conversion of “cold” tumors improves the efficacy of cancer immunotherapy.^252^ The change of the relationship between tumor cells and immune cells leads to the process of cancer occurrence, development, invasion, and metastasis. TME, immune functions, organ metabolism mechanism, and signaling pathway are closely related to cancer development. Focusing on the changes of tumor immune microenvironment and immune function are the keys to improving the efficacy and survival rate of immunotherapy in advanced cancer patients.^253^ A thorough examination of tumor tissue, blood samples, and clinical data of the host utilizing diverse diagnostic methodologies such as molecular, cellular, histological, imaging, and artificial intelligence can facilitate the identification of specific immunological characteristics of patients.^254^, ^255^, ^256^, ^257^ This approach represents a novel avenue for cancer immunotherapy, aimed at converting “cold” tumors into “hot” tumors, thereby creating a conducive environment for enhancing the effectiveness of immunotherapy, which has great potential for clinical application.

Although great progress has been made in recent years, limited by the spatial distribution of immune cells, cold tumors are often “insensitive” to immunotherapy, and ICI treatment is still limited in the treatment of cold and altered tumors. There is still a lot to learn about how to overcome the limitations of ICI in cold tumor therapy. Local immunomodulatory therapy, such as elevating the expression of tumor antigens, restoring antigen processing and presentation, and reprogramming TME, can promote T cell transport and enable T cells to infiltrate tumor more effectively, resulting in synergistic effect with ICI.^258^ However, the existence of immune response and immune drug resistance is likely to limit the efficacy of a single ICI due to tumor‐mediated immunosuppression. The colder the tumor, the more it requires a combination of therapies to achieve significant results.

Multitargets drug therapy may represent the future of “hotness and coldness tumor” immunotherapy strategies. From the current situation, molecular drugs represented by targeted therapies and immunotherapies are important parts of cancer research. Targeted drugs prevent the growth of cancer cells by interacting with specific molecular targets necessary for cancer occurrence and tumor growth, while immunotherapy restarts and maintains the tumor‐immune cycle to restore the body's normal antitumor immune response, control and eliminate cancer cells. However, the mechanisms of cancer occurrence and progression are very complex, more than one related gene is involved, often rely on a variety of signaling pathways to maintain growth and survival, and there is crossover and compensation between signaling pathways, only inhibiting one (or the same type of) target has limited effect. For cancer patients, targeted therapy or immunotherapy that has a single‐target is prone to drug resistance, thus affecting the long‐term efficacy of cancer treatment, and multitarget therapy that inhibits multiple signaling pathways or multiple molecules in downstream of a pathway to achieve simultaneous treatment can effectively plan for this shortcoming. Therefore, the future research direction of “coldness and hotness tumor” is to combine the advantages of targeted therapy and immunotherapy, develop a single drug target to a multitarget direction, and enhance the immune response of “coldness tumor” effectively.

Targeted therapies for the precise treatment of cancer based on genetic mutations and Immunotherapy for individualized cancer treatment based on TME and tumor heterogeneity provide a variety of cancer therapies for the research of “hotness and coldness tumors.” Now we can fortunately see that under the precision and personalized combined treatment, some malignant tumors have become a slowly progressing disease, patients can survive for a long time, and malignant tumors have entered the era of “chronic disease.” With the existing drugs, it is very necessary to develop multitarget drugs that transform “coldness tumor” into “hotness tumor” to meet clinical needs. The natural drugs recorded in TCM may be the best treasure house to search for multitarget drugs. Whether it is plant drugs, animal drugs or mineral drugs, the rich and effective drug components provide important data for the research of multitarget drugs for cancer treatment.

## AUTHOR CONTRIBUTIONS

Lianjie Wang, Hui Geng, and Yujie Liu contributed to the conception of the study and wrote the initial draft. Lei Liu, Yanhua Chen, Fanchen Wu, and Zhiyi Liu reviewed the manuscript and provided suggestions for revision. Shiliang Ling, Yan Wang, and Lihong Zhou helped perform the analysis with constructive discussions. All authors read and approved the final manuscript.

## CONFLICT OF INTEREST STATEMENT

The authors declare that there are no conflicts of interest regarding the publication of this paper.

### ETHICS STATEMENT

Not applicable.

## Data Availability

Not applicable.
